# The protective effects of dietary microalgae against hematological, biochemical, and histopathological alterations in pyrogallol-intoxicated *Clarias gariepinus*

**DOI:** 10.1016/j.heliyon.2024.e40930

**Published:** 2024-12-05

**Authors:** Mohamed Hamed, Nasser S. Abou Khalil, Alshaimaa A.I. Alghriany, Alaa El-Din H. Sayed

**Affiliations:** a Department of Comparative Biomedical Sciences, School of Veterinary Medicine, Louisiana State University, Skip Bertman Drive, Baton Rouge, LA, 70803, USA; b Department of Zoology, Faculty of Science, Al-Azhar University (Assiut branch), Assiut, 71524, Egypt; c Department of Medical Physiology, Faculty of Medicine, Assiut University, Assiut, Egypt; d Department of Animal Physiology and Biochemistry, Faculty of veterinary Medicine, Badr University, Assuit, Egypt; e Department of Zoology, Faculty of Science, Assiut University, Assiut, 71516, Egypt; f Molecular Biology Research & Studies Institute, Assiut University, 71516, Assiut, Egypt

**Keywords:** Fish, Microalgae, Pyrogallol, Hematology, Biochemistry, Histopathology

## Abstract

Microalgae have well-established health benefits for farmed fish. Thus, this study aims to explore the potential protective effects of *Spirulina platensis*, *Chlorella vulgar*is, and *Moringa oleifera* against pyrogallol-induced hematological, hepatic, and renal biomarkers in African catfish (*Clarias gariepinus*), as well as the histopathological changes in the liver and kidney. Fish weighing 200 ± 25 g were divided into several groups: group 1 served as the control, group 2 was exposed to 10 mg/L of pyrogallol, and groups 3, 4, and 5 were exposed to the same concentration of pyrogallol, supplemented with *S. platensis* at 20 g/kg diet, *C. vulgar*is at 50 g/kg diet, and *M. oleifera* at 5 g/kg diet, respectively, for 15 days. Exposure to pyrogallol led to decreased packed cell volume (PCV) and lymphocyte count, but these effects were alleviated by microalgae interventions. *C. vulgar*is and *M. oleifera* equally restored PCV and increased lymphocyte counts. Supplementation with *C. vulgar*is and *M. oleifera* successfully normalized both neutrophil and eosinophil counts. Pyrogallol intoxication engenders an increase in glycemic status, but *C. vulgar*is and *M. oleifera* effectively mitigated this rise. Pyrogallol-exposed fish exhibited signs of renal dysfunction, with increased serum creatinine and total cholesterol levels. A significant decrease in both erythrocytic cellular and nuclear abnormalities was observed following supplementation with microalgae. *C. vulgaris* and *M. oleifera* showed promise in decreasing serum glucose and creatinine levels, and improving hematological parameters, while *S. platensis* exhibited limited efficacy in this regard. Exposure to pyrogallol led to a notable decrease in serum superoxide dismutase activity and total antioxidant capacity (TAC), accompanied by an increase in serum malondialdehyde (MDA) levels. Diets enriched with *C. vulgaris* and *M. oleifera* effectively restored these parameters to normal levels, whereas *S. platensis* did not induce significant changes. None of the microalgae improved TAC except for *M. oleifera*, which significantly enhanced it. MDA levels returned to control levels equally and significantly across all groups. Interleukin-6 levels did not exhibit significant differences between any of the groups. Collectively, the histopathological changes induced by pyrogallol were most prominently alleviated in the pyrogallol + *C. vulgar*is and pyrogallol + *M. oleifera* groups, and to a limited degree in the pyrogallol + *S. platensis* group. While the tested microalgae did not cause hepatic or renal dysfunction, they did lead to metabolic abnormalities. The incorporation of microalgae into the diet holds significant importance in mitigating the metabolic and histological toxicity of pyrogallol and should be considered in the formulation of fish feed.

## Highlights

• Pyrogallol presents a risk to aquatic ecosystems.
• Pyrogallol intoxication results in increased serum creatinine levels and metabolic abnormalities.
• *Spirulina platensis, Chlorella vulgaris,* and *Moringa oleifera* mitigate pyrogallol-induced adverse effects.
• Microalgae alleviate histopathological changes induced by pyrogallol in the liver and kidney.

## 1 Introduction

Environmental pollutants include industrial and agricultural chemicals, heavy metals, pharmaceutical agents, as well as products with hormonal activity [1]. They lead to interruptions in the reproductive endocrine circuits, cytotoxicity, mutagenicity [1], growth arrest, hemato-biochemical deteriorations, immunosuppression, and redox imbalance [2] in fish. Still further, these contaminants accumulate in fish tissues, potentially harming human well-being through the food chain [3]. Pyrogallol, alternatively termed 1,2,3-trihydroxybenzene or 1,2,3-benzenetriol, is a phenolic compound derived from plants [4]. It has a historical background in hair dyeing and continues to have diverse applications in modern industry [5]. Its uses range from being a corrosion inhibitor and developer in holography and photography to being present in insecticides, colloidal metal solutions, and various medical and scientific products [6]. Pyrogallol is naturally found in aquatic plant [7] and as a contaminant in tannins, anthocyanins, flavones and alkaloids and released into environment during its isolation, disposal and industrial use [8].

Pyrogallol improved the immune response by increasing myeloperoxidase activity, leukocyte respiratory burst activity, and lysozyme activity in zebrafish (*zebra danio*) against *Acinetobacter baumannii* infection [9]. Pretreating brine shrimp (*Artemia franciscana*) with pyrogallol succeeded in counteracting *Vibrio harveyi* infection by its prooxidant action involving generation of hydrogen peroxide against [10].

Microalgae are promising ingredients for feed sources due to their cellular metabolites comprising a blend of triglycerides, pigments, vitamins, and vital amino acids. Besides being a major component in aquafeed, their diverse range of biologically active ingredients can enhance the survival rates of farmed species and elevate the pigmentation and quality of edible meat [[11], [12], [13], [14], [15]]. Along with their role as a cornerstone in aquaculture nutrition, they are highly valuable in combating ecotoxicity [16].

*Spirulina platensis*, *Chlorella vulgaris*, and *Moringa oleifera* serve as effective interventions in mitigating the harmful effects of environmental contaminants on fish, and thereby potentially improving their health and overall well-being. These supplements could ameliorate hematological, hepatic, renal, and histopathological alterations caused by different toxicants, such as chlorpyrifos, diazinon, and oxyfluorfen [17,18].

Haematological outcomes provide straightforward and convincing methodological tools to comprehend the physiological processes and diagnose the somatic conditions of intoxicated fish [19]. They identify anomalies within individuals even before observable negative effects on the ecosystem were noted [20]. Despite various morphological, biochemical, and molecular analysis for evaluating the health status of fish, histopathologic evaluation remains a commonly employed technique in ecotoxicological investigations [21].

*C. gariepinus* holds a prominent position as a widely favored freshwater fish globally, playing a crucial role as a significant nutritional source and a thriving species [22] In terms of ecotoxicology, *C. gariepinus* is highly regarded as an outstanding model for evaluating the plausible harm of chemotoxicants, both within natural environments and controlled laboratory setups [22,23]. As an indispensable source of human food, the toxicity in *C. gariepinus* could be transferred to human beings through the food chain. Therefore, the search for naturally occurring bioremediation strategies, with broad safety profiles, is of immense importance. These methods play a significant role not only in protecting the aquatic environment but also in ensuring human health. Upon thorough examination of scholarly articles, a notable gap exists in understanding the defensive properties of microalgae against toxicity triggered by pyrogallol in fish. Consequently, this study designs to address this gap by investigating hematological, biochemical, and histological markers as endpoints to explore this issue.

## 2 Materials and methods

### 2.1 Chemicals

Pyrogallol, acquired in the form of solid white crystals, was obtained from Sigma-Aldrich Chemical Company (USA). Its specifications include a chemical formula of C6H3(OH)3, quality level 200, a molecular weight of 126.11 g/mol, and a Chemical Abstracts Service number of 87-66-1. *S. platensis* (100 %) was sourced from Japan Algae Company, based in Tokyo, Japan, while *C. vulgaris* and *M. oleifera* were acquired from Sigma-Aldrich (Cairo, Egypt)

### 2.2 Experimental protocol

The animal study conducted in this research underwent thorough review and received approval from Al-Azhar University, Assiut Branch, Egypt. The Ethical Committee of the Faculty of Science at Al-Azhar University, Assiut Branch, granted approval for the experimental design and fish handling protocols, referencing approval number (AZHAR 10/2022). A total of 150 *C. gariepinus*, weighing approximately 200 ± 25 g and measuring 25 ± 5 cm in length, were sourced from the Aquaponic Unit of Assuit University, Assuit, Egypt. Before the experiments, the fish were housed in 120-liter tanks for 14 days, where they were provided with dechlorinated tap water at a temperature of 26 ± 2 °C, pH levels between 7.2 and 7.6, oxygen levels maintained above 80 %, and a natural light cycle of 12 h each of light and dark. They were fed twice daily using commercial SKRETTING food formulated in line with Guideline No. 203 for testing the acute toxicity of chemicals in fish (OECD, 2019), containing 30 % protein. To minimize the impact of fish waste and ensure consistency, daily, 40 % of the water was exchanged. After the adaptation period, fifty fish were separated in a random fashion into five groups (30 fish per group). Each group was represented by three tanks, 10 fish each. Pyrogallol concentration was selected based on a previous research [22]. The experimental groups were as follows: Group (1); control, Group (2); 10 mg/L of pyrogallol, Group (3); 10 mg/L of pyrogallol + *S. platensis* (20 g/kg diet) [24], Group (4); 10 mg/L of pyrogallol + *C. vulgar*is (50 g/kg diet) [25], Group (5); 10 mg/L of pyrogallol + *M. oleifera* (5 g/kg diet) [26]. After being exposed for 15 days, six fish were chosen randomly and then subjected to an ice bath to relieve stress (Wilson et al., 2009). Blood samples were gathered in tubes containing heparin for hematological evaluations, while non-heparinized tubes were employed for assessing biochemical markers and ions. This was done after tail cutting to collect blood from the caudal blood vessels.

### 2.3 Hematological variables

The red blood cell count (RBC, 10^6/mm^3) was determined using a Thoma hemocytometer chamber and Dacie's diluting fluid. The hematocrit ratio (Hct, %) was measured with a capillary hematocrit tube. Hemoglobin concentration (Hb, g/dL) was assessed through spectrophotometry at 540 nm utilizing the cyanomethemoglobin method described by Blaxhall and Daisley (1973). Mean corpuscular volume (MCV), mean corpuscular hemoglobin (MCH), and mean corpuscular hemoglobin concentration (MCHC) were computed using formulas provided by Bain et al. (2016).

Peripheral blood smears stained with May-Grunwald-Giemsa were examined at × 100 oil immersion to analyze differential leukocytes. The count of white blood cells (WBC, 10^3^/mm^3^) was determined following McKnight's protocol from 100 leukocytes on each slide to identify lymphocytes, neutrophils, and monocytes, as detailed by Kaya et al. [27].

### 2.4 Morphological erythrocytic alterations and nuclear abnormalities

After the exposure phase, blood smears were attained by making an incision in the caudal area and placing the blood on clean microscopic slides. These smears were then fixed in absolute methanol for 10 min and allowed to dry at room temperature. Afterward, the slides underwent staining with hematoxylin and eosin, followed by examination using a 40X objective lens and 10X eyepiece to detect micronucleated and morphologically changed RBCs. The identification requirements for micronuclei (MN) were based on established guidelines from prior studies [28] to guarantee precise evaluation.

### 2.5 Biochemical measurements

Blood samples were collected and then centrifuged at 4000 rpm for 10 min to separate the serum, following [29]. The isolated serum was analyzed for different biochemical parameters using a T80+ UV/VIS spectrophotometer (Bioanalytic Diagnostic Industry, Co.). Plasma alanine aminotransferase (ALT) (Catalog number: 264001), aspartate aminotransferase (AST) (Catalog number: 260001), total protein (Catalog number: 310001), glucose (Catalog number: 250001), total cholesterol (TC) (Catalog number: 230002), and creatinine (Catalog number: 235001) were assessed using commercial kits from the Egyptian Company for Biotechnology, Egypt, according to the manufacturer's instructions. Alkaline phosphatase (ALP) (Catalog number: NBP3-24466) was measured using a colorimetric kit from Novus Biologicals, USA, and uric acid (UA) (Catalog number: KA1651) was measured using a kit from Abnova, Taiwan. All these biochemical parameters were analyzed according to Rifai [30]. Malondialdehyde (MDA) was quantified in serum according to the thiobarbituric acid reaction [31]. Superoxide dismutase (SOD) activity was measured by its capacity to inhibit the phenazine methosulfate-mediated reduction of nitroblue tetrazolium dye, which produces a red product [32]. Total antioxidant capacity (TAC) was determined following the protocol outlined by Koracevic et al. (2001). Levels of interleukin-6 (IL-6) were measured according to an established method [33].

### 2.6 Histopathological studies

Samples of freshly sacrificed fish liver and kidney (N = 6) were immersed in 10 % neutral buffered formalin for preservation. The fixed samples underwent standard processing using paraffin embedding techniques. Subsequently, the samples were sliced into 5 μm-thick sections and stained with Hematoxylin and Eosin for comprehensive histological investigation (Robert et al., 2014), and Periodic Acid Schiff (PAS) staining was used to detect mucopolysaccharides (McManus, 1946).

To assess each histopathological parameter, six randomly chosen sections from four fish in each treatment group were examined. These parameters were categorized as follows: absent (-), scored 0-2%; slight (+), <25%; moderate (++), (25-50%); and severe (+++), indicating (> 50%) of the sections involved. An Olympus microscope model BX50F4 (Olympus Optical Co., Ltd., Tokyo, Japan) was used for scrutiny of the sections.

### 2.7 Statistical analysis

Six biological replicates for each parameter outline above were statistically processed using the SPSS software (V 25) at a designated significance level of p < 0.05. Data were tested for normality using the Shapiro-Wilk test and for homogeneity of variance to ensure that the data were normally distributed, and the variances were consistent. One-way ANOVA followed by Duncan post-test. Data are depicted as mean ± SE. For each parameter, values in the same row with distinct superscript letters (a, b, c …) indicate a difference among groups.

### Ethics approval

The animal research conducted at Al-Azhar University, Assiut Branch, Egypt received approval from the institution. The experimental setup and treatment of the fish were sanctioned by the Ethical Committee of the Faculty of Science, Al-Azhar University, Assiut Branch, Egypt, referenced as (AZHAR 10/2022). This research did not involve human participants and was conducted following international, national, and institutional standards for the care and use of animals.

## 3 Results

### 3.1 Hematological variables

Regarding hematological parameters, pyrogallol-exposed fish experienced a significant decrease in PCV and lymphocyte count, which was significantly ameliorated by microalgae interventions. *C. vulgar*is and *M. oleifera* were equally effective in normalizing PCV levels and significantly increasing lymphocyte counts. Neutrophil and eosinophil counts were significantly elevated in pyrogallol-exposed fish, and while *S. platensis* didn't significantly affect neutrophil levels, it significantly reduced eosinophil counts. *C. vulgar*is and *M. oleifera* supplementation significantly normalized these levels to those of the control group (Table 1).

**Table 1:** Effects of the studied microalgae on the hematological parameters in pyrogallol intoxicated-*C. gariepinus.*

Parameter	Group					
	Control	Pyrogallol	Pyrogallol + *S. platensis*	Pyrogallol + *C. vulgaris*	Pyrogallol + *M. oleifera*	P value
**RBCs (million/mm**^**3**^**)**	3.17 ± 0.07	2.75 ± 0.05	3.17 ± 0.24	3.03 ± 0.07	3.19 ± 0.09	0.104
**Hb (g/dL)**	8.94 ± 0.49	7.85 ± 0.45	8.32 ± 0.21	8.23 ± 0.20	9.49 ± 0.50	0.070
**PCV (%)**	35.28 ± 0.31	32.33 ± 0.29^Φ^	33.57 ± 0.35^Ω^	34.21 ± 0.58^Ω^	35.34 ± 0.33^Ω^	0.000
**MCV (μm**^**3**^**)**	109.48 ± 1.56	116.60 ± 2.50	106.63 ± 7.90	111.91 ± 2.43	109.99 ± 2.14	0.522
**MCH (pg)**	27.67 ± 0.98	28.24 ± 1.35	26.32 ± 1.64	26.92 ± 0.85	29.42 ± 0.81	0.417
**MCHC (%)**	25.00 ± 1.16	24.02 ± 1.31	24.54 ± 0.64	23.80 ± 0.27	26.55 ± 1.19	0.353
**Platelet count ( × 10**^**3**^**/mm**^**3**^**)**	216.53 ± 5.40	205.92 ± 2.68	211.61 ± 4.87	214.48 ± 1.52	222.02 ± 5.48	0.160
**WBCs count ( × 10**^**3**^**/mm**^**3**^**)**	11.67 ± 0.21	10.74 ± 0.20	11.24 ± 0.28	11.63 ± 0.25	11.50 ± 0.39	0.160
**Large lymphocytes (%)**	57.77 ± 0.23	54.45 ± 0.70^Φ^	55.69 ± 0.62	56.89 ± 0.74^Ω^	57.10 ± 0.51^Ω^	0.010
**Small lymphocytes (%)**	24.20 ± 0.32	21.53 ± 0.25^Φ^	23.02 ± 0.47^Ω^	23.89 ± 0.29^Ω^	24.75 ± 0.35^Ω^	0.000
**Neutrophils (%)**	11.11 ± 0.26	13.86 ± 0.57^Φ^	13.12 ± 0.47	12.06 ± 0.70^Ω^	11.27 ± 0.29^Ω^	0.005
**Monocytes (%)**	2.96 ± 0.01	1.98 ± 0.40	2.23 ± 0.25	2.47 ± 0.29	2.69 ± 0.24	0.144
**Eosinophils (%)**	2.72 ± 0.25	7.18 ± 0.47^Φ^	4.70 ± 0.85^Ω^	3.20 ± 0.24^Ω^	2.20 ± 0.24^Ω^	0.000

RBCs: red blood cells; Hb: hemoglobin; PCV: packed cell volume; MCV: mean corpuscular volume; MCH: mean corpuscular hemoglobin; MCHC: mean corpuscular hemoglobin concentration; WBCs: white blood cells. Results are expressed as the mean ± SEM of 6 fish per group (One-way ANOVA followed by Duncan post-test). ^Φ^ Significant difference at p < 0.05 between control and pyrogallol groups; ^Ω^ Significant difference at p < 0.05 between pyrogallol and microalgae groups.

### 3.2 Morphological erythrocytic alterations and nuclear abnormalities

**Fig. 1:**
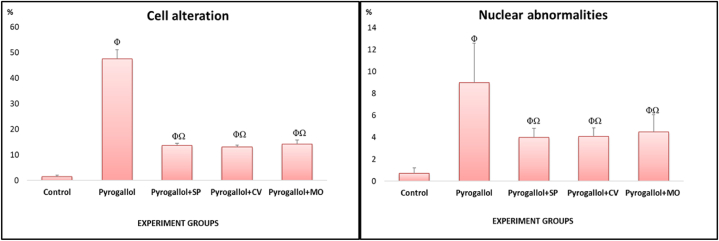
The percentage of cell alterations and nuclear abnormalities in RBCs of pyrogallol-intoxicated *C. gariepinus* after 15 days of treatment with *S. platensis* (SP), *C. vulgaris* (CV), and *M. oleifera* (MO). Results are expressed as the mean ± SEM of 6 fish per group (One-way ANOVA followed by Duncan post-test). Φ Significant difference at p < 0.05 between control and pyrogallol groups; Ω Significant difference at p < 0.05 between pyrogallol and microalgae groups.

**Fig. 2:**
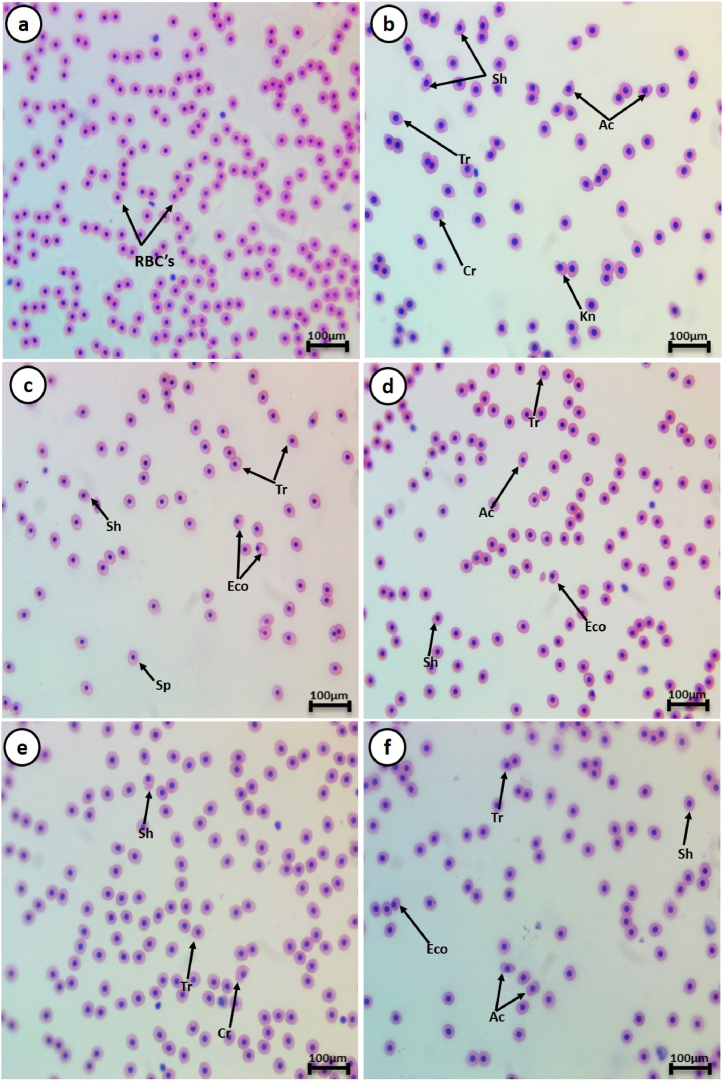
Represented blood smears in *C. gariepinus* showing (a) the normal erythrocytes, (b and c) the deformed ones after exposure to 10 mg/L pyrogallol, (d) the deformed ones after exposure to 10 mg/L of pyrogallol + *S. platensis* (20 g/kg diet), (e) 10 mg/L of pyrogallol + *C. vulgaris* (50 g/kg diet) and (f) 10 mg/L of pyrogallol + *M. oleifera* (5 g/kg diet); Tr, tear-drop cell; Sp, spinocyte; Cr, crenated cell; Ac, acanthocyte; Eco, ecocentric nucleus; Bn, Bionucleus; Kn, kidney shape and Sh, schistocytic (H & E stain, scale bar: 100 μm).

In the control group, RBCs displayed a typical shape with a nucleus positioned centrally. However, smears from the pyrogallol group exhibited poikilocytosis in RBCs after the exposure periods. Various morphological changes were evident, including tear-drop cells, spinocytes, crenated cells, acanthocytes, eccentric nuclei, kidney-shaped cells, and schistocytes (Fig. 1, Fig. 2). The frequency of cell modifications and nuclear irregularities in RBCs notably rose in the intoxicated group in comparison to the control group (Fig. 1).

Upon a 15-day exposure to 10 m/L of pyrogallol alone, the observed alteration percentages were 47.5 ± 3.59 % for cell alterations and 9 ± 1.41 % for nuclear abnormalities compared to the control group. Nevertheless, following the same exposure duration, the observed alteration percentages were 13.7 ± 0.82 %, 13.1 ± 0.73 %, and 14.2 ± 1.54 % for cell alterations, and 4 ± 0.81 %, 4.1 ± 0.8 %, and 4.5 ± 0.7 % for nuclear abnormalities when exposed to 10 mg/L pyrogallol + *S. platensis*, 10 mg/L of pyrogallol + *C. vulgar*is, and 10 mg/L of pyrogallol + *M. oleifera*, respectively. Thus, the supplementation of pyrogallol-exposed groups with *S. platensis*, *C. vulgar*is, and *M. oleifera* showed a significant reduction in cellular and nuclear abnormalities.

### 3.3 Hepatic and renal damage biomarkers

The pyrogallol-intoxicated groups showed a significant increase in blood glucose levels compared to the control. However, treatments with *C. vulgar*is and *M. oleifera* significantly reduced these elevated glucose levels, while SP did not yield significant changes. Pyrogallol-exposed fish displayed increased serum creatinine and TC levels. Yet, all studied microalgae significantly decreased these elevated levels. *M. oleifera* exhibited superior efficacy compared to *S. platensis* in reducing serum creatinine and TC levels. Remarkably, there were no significant alterations in serum AST, ALT, and TP between the groups (Table 2).

**Table 2:** Effects of the studied microalgae on hepatic and renal damage biomarkers in pyrogallol intoxicated-*C. gariepinus.*

Parameter	Group					
	Control	Pyrogallol	Pyrogallol + *S. platensis*	Pyrogallol + *C. vulgaris*	Pyrogallol + *M. oleifera*	P value
**AST activity (μ/l)**	34.89 ± 1.06	35.33 ± 0.78	34.80 ± 0.89	34.41 ± 0.88	32.58 ± 0.73	0.253
**ALT activity (μ/l)**	17.23 ± 0.53	18.09 ± 0.54	17.48 ± 0.44	17.14 ± 0.51	16.57 ± 0.53	0.364
**ALP activity (μ/l)**	47.40 ± 1.70	43.31 ± 1.36	45.29 ± 1.48	46.07 ± 2.11	44.58 ± 1.74	0.532
**Glucose level (mg/dl)**	70.22 ± 1.61	78.61 ± 0.45^Φ^	74.18 ± 2.15	72.91 ± 1.68^Ω^	72.39 ± 1.01^Ω^	0.016
**TP level (mg/dl)**	3.98 ± 0.16	4.56 ± 0.28	4.33 ± 0.20	4.21 ± 0.23	4.17 ± 0.17	0.441
**TC level (mg/dl)**	210.09 ± 0.63	223.25 ± 3.30^Φ^	215.57 ± 1.42^Ω^	212.75 ± 1.62^Ω^	208.29 ± 1.89^Ω^	0.000
**Creatinine level (mg/dl)**	0.36 ± 0.01	0.46 ± 0.02^Φ^	0.39 ± 0.01^Ω^	0.38 ± 0.01^Ω^	0.35 ± 0.01^Ω^	0.000
**UA level (mg/dl)**	22.84 ± 0.25	23.27 ± 0.37	22.67 ± 0.12	22.73 ± 0.23	22.03 ± 0.21^Ω^	0.043

AST: aspartate aminotransferase; ALT: alanine transaminase; ALP: alkaline phosphatase; TP: total protein; TC: total cholesterol; UA: uric acid. Results are expressed as the mean ± SEM of 6 fish per group (One-way ANOVA followed by Duncan post-test). ^Φ^ Significant difference at p < 0.05 between control and pyrogallol groups; ^Ω^ Significant difference at p < 0.05 between pyrogallol and microalgae groups.

### 3.4 Oxidative stress parameters and pro-inflammatory interleukin-6

Pyrogallol exposure resulted in a significant reduction in serum SOD activity and TAC, along with an elevation in serum MDA levels. Diets enriched with *C. vulgaris* and *M. oleifera* significantly normalized these parameters, while *S. platensis* did not produce any significant changes. All microalgae failed to improve TAC except for *M. oleifera*, which significantly increased it. MDA levels returned to the control level equally and significantly. IL-6 levels did not show significant changes between any of the groups (Table 3).

**Table 3:** Effects of the studied microalgae on serum oxidative stress parameters and pro-inflammatory interleukin-6 in pyrogallol intoxicated-*C. gariepinus.*

Parameter	Group					
	Control	Pyrogallol	Pyrogallol + *S. platensis*	Pyrogallol + *C. vulgaris*	Pyrogallol + *M. oleifera*	P value
**SOD activity (U/ml)**	2.68 ± 0.06	1.91 ± 0.10^Φ^	2.23 ± 0.13	2.63 ± 0.19^Ω^	2.67 ± 0.05^Ω^	0.000
**TAC (nmol/l)**	56.86 ± 4.28	42.65 ± 1.16^Φ^	43.76 ± 0.40	48.61 ± 3.10	50.40 ± 1.20	0.007
**MDA level (nmol/ml)**	14.94 ± 1.28	30.57 ± 2.60^Φ^	17.97 ± 0.39^Ω^	16.59 ± 0.95^Ω^	16.87 ± 1.37^Ω^	0.000
**IL-6 level (pg/ml)**	51.15 ± 0.34	53.42 ± 0.23^Φ^	55.77 ± 0.47	58.04 ± 1.75	55.72 ± 2.91	0.060

SOD: superoxide dismutase; TAC: total antioxidant capacity; MDA: malondialdehyde; IL-6: interleukin-6. Results are expressed as the mean ± SEM of 6 fish per group (One-way ANOVA followed by Duncan post-test). ^Φ^ Significant difference at p < 0.05 between control and pyrogallol groups; ^Ω^ Significant difference at p < 0.05 between pyrogallol and microalgae groups.

### 3.5 Histopathological and histochemical findings

**Fig. 3:**
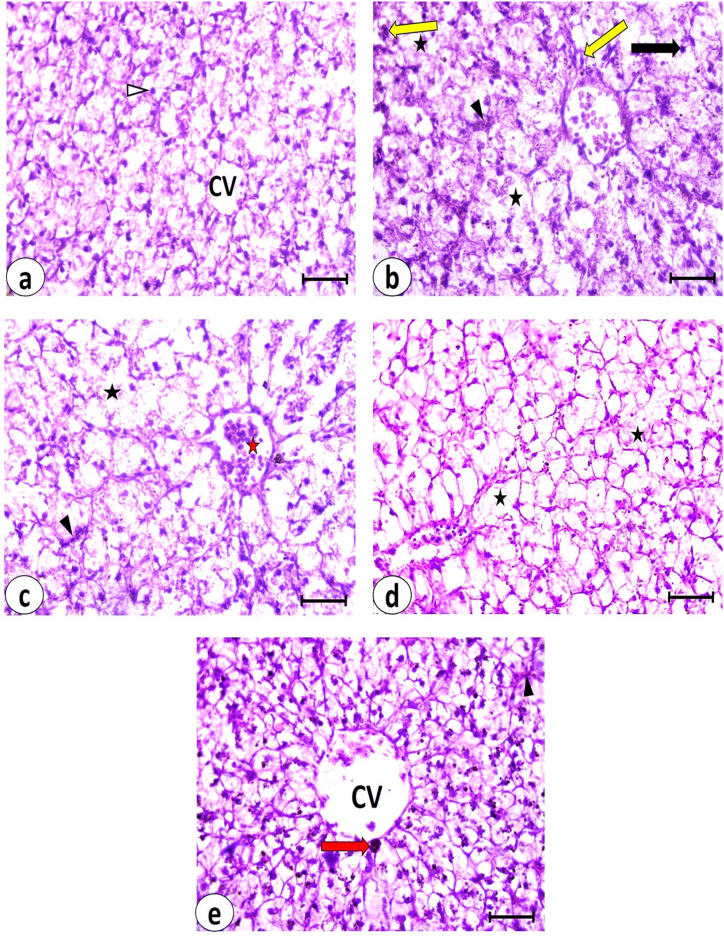
Photomicrographs of liver sections in *C. gariepinus* stained by H&E (bars = 50 μm) showing: **(a)** control group, **(b)** Pyrogallol group, **(c)** Pyrogallol + *S. platensis*, **(d)** Pyrogallol + *C. vulgaris* and **(e)** Pyrogallol + *M. oleifera*; CV, **central vein**; **Δ**, rounded vesicular nucleus of hepatocyte; ▲, congested blood sinusoid; **↑**, pyknotic nucleus; **black asterisk**, necrotic area; **yellow arrow**, inflammatory cells infiltration, **red asterisk**, congested central vein and **red arrow**, pigments.

Liver sections stained with Hematoxylin and Eosin from the control group (Fig. 3a) showed a normal structure. The hepatocytes appeared polygonal with unstained cytoplasm and centrally or eccentrically located rounded vesicular nuclei. Cords of hepatocytes radiated from central veins with blood sinusoids located between these cords. In the pyrogallol group, hepatic tissue exhibited numerous necrotic areas, pycnotic nuclei of hepatocytes, and congested dilated blood sinusoids (Fig. 3b). Inflammatory cell infiltration was observed. Mild improvement was detected in liver sections from the pyrogallol + *S. platensis* group (Fig. 3c). There was congestion of central veins and blood sinusoids, and pycnotic nuclei of some hepatocytes were noted. Necrotic areas were dispersed throughout the hepatic tissue. The liver of the pyrogallol + *C. vulgaris* group (Fig. 3d) showed moderate improvement compared to the pyrogallol group, with some hepatocytes resembling those of the control group. Necrotic areas were still clearly observed. Marked improvement was observed in the hepatic tissue of the pyrogallol + *M. oleifera* group (Fig. 3e). The hepatocytes closely resembled those of the control group, although some blood sinusoids remained congested. Pigments were observed near the central vein. The histopathological scoring of lesions observed in the hepatic tissue is represented in Table 4.

**Table 4:** Scoring of histopathological lesions in the liver and kidney of the examined groups.

Lesions	Groups				
	Control	Pyrogallol	Pyrogallol + *S. platensis*	Pyrogallol + *C. vulgaris*	Pyrogallol + *M. oleifera*
Liver					
**Hepatocytes with pycnotic nuclei**	–	+++	+++	+	++
**Necrotic areas**	–	+++	++	++	+
**Inflammatory cells infiltration**	–	+++	++	+	+
**Congestion of blood vessels**	–	++	+++	+	+
**Dilated blood sinusoids**	–	++	++	+	+
**Hemorrhage**	–	+	+	–	–
**Kidneys**					
**Bowman's capsule thickening**	–	+++	+++	+	–
**Glomeruli shrinkage**	–	++	+	+	–
**Glomerular tuft vacuolar degeneration**	+	++	+	–	+
**Complete degeneration of renal tubules**	–	+++	++	+	–
**Separation of tubular cells from basement membrane**	+	+++	++	+	+
**Pyknotic nuclei of renal tubular cells**	+	+++	++	+	+
**Vacuolated renal tubular cells**	–	+++	++	+	+
**Inflammatory cells infiltration**	+	++	++	+	+
**Melanomacrophage centers**	+	+++	++	++	++
**Hematopoietic tissue**	+	+++	+++	++	+

(−) Absent, **(+)** Slight (<25 %), **(++)** Moderate (from 25 to 50 %), and **(+++)** Severe lesion (>50 %).

**Fig. 4:**
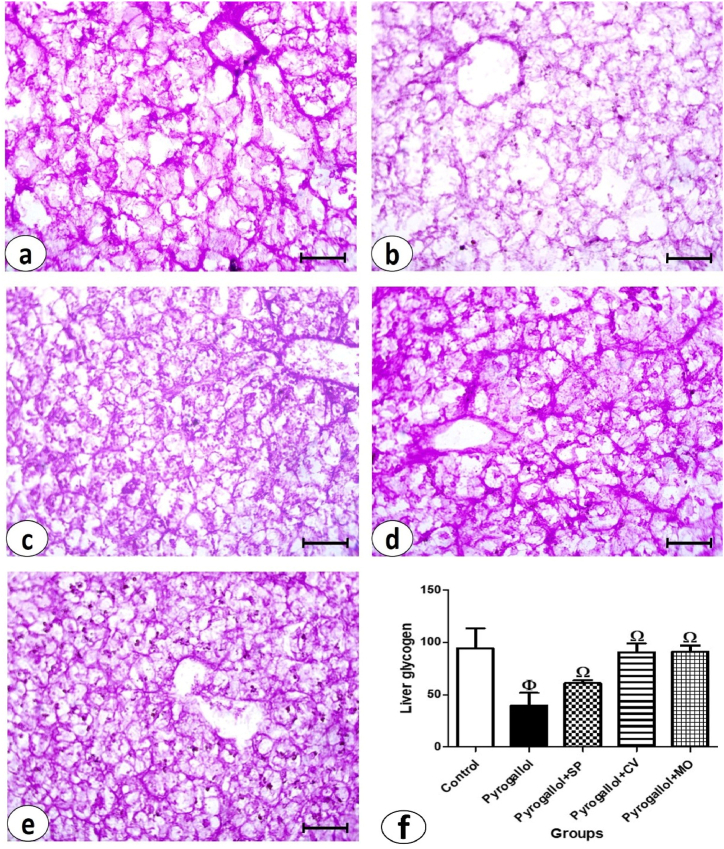
Examination of liver glycogen in *C. gariepinus*. **(a**–**e)**: photomicrographs of liver sections stained by PAS stain (bars = 50 μm) showing: **(a)** control group with normal glycogen content as represented by magenta color, **(b)** Pyrogallol group, **(c)** Pyrogallol + *S. platensis* (SP), **(d)** Pyrogallol + *C. vulgaris* (CV), and **(e)** Pyrogallol + *M. oleifera* (MO). **(f)** liver glycogen in sections from all experimental groups. Bars represent means ± SE. Different letters indicate significant differences among treatments (p < 0.5).

The liver sections stained with PAS staining revealed the glycogen content in the hepatic tissue (Fig. 2). The magenta color observed in the liver sections from the control group indicated a normal amount of glycogen (Fig. 4a). A significant reduction in glycogen levels was observed in the pyrogallol-treated group (Fig. 4b), which was statistically significant compared to the control group (Fig. 4f). Glycogen content increased significantly in the pyrogallol + *C. vulgaris* and pyrogallol + *M. oleifera* groups compared to the pyrogallol group, but not significantly in the pyrogallol + *S. platensis* group. There were no significant differences in glycogen content among the three treated groups (pyrogallol + *S. platensis*, pyrogallol + *C. vulgaris*, and pyrogallol + *M. oleifera*) and the control group (Fig. 4c, d, e & f).

**Fig. 5:**
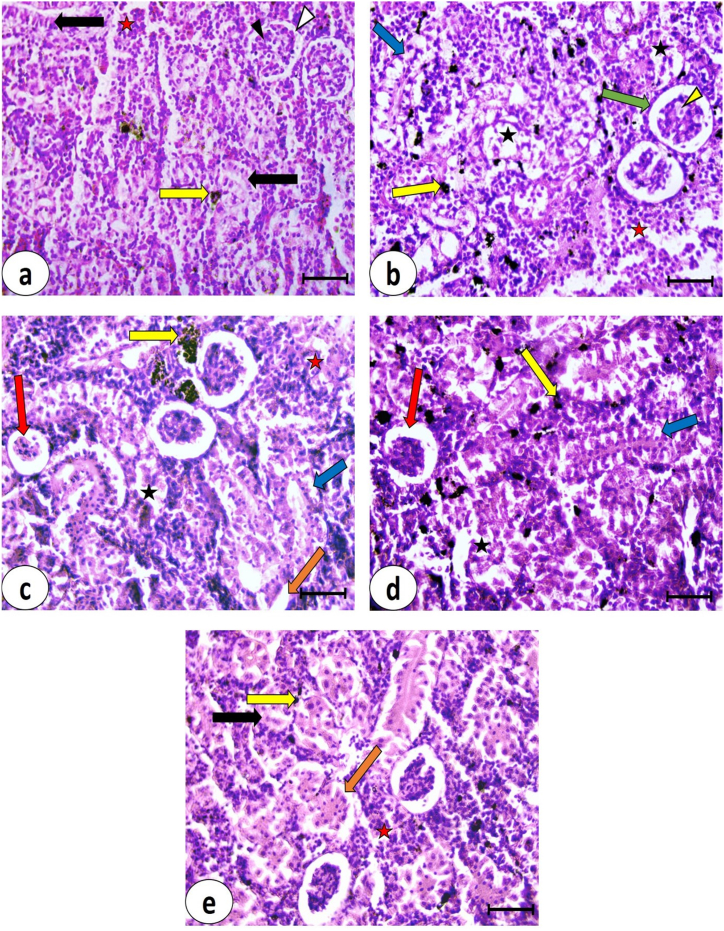
Photomicrographs of kidney sections in *C. gariepinus* stained by H&E (bars = 50 μm) showing: **(a)** control group, **(b)** Pyrogallol group, **(c)** Pyrogallol + *S. platensis*, **(d)** Pyrogallol + *C. vulgaris* and **(e)** Pyrogallol + *M. oleifera*; Δ, Bowman's capsule; ▲, glomerulus; **↑**, renal tubular cell; **yellow arrow**, melanomacrophage; **red asterisk**, hematopoietic tissue; **black asterisk**, necrotic area; **green arrow**, thickening of Bowman's capsule; **yellow arrowhead**, glomerular vacuolar degeneration; **blue arrow**, vacuolated renal tubular cell with pyknotic nucleus; **red arrow**, shrunken glomerulus and **orange arrow**, separation of renal tubular cells from basement membrane.

The kidney of the control group (Fig. 5a) showed Malpighian corpuscles with Bowman's space separating glomerular tufts and Bowman's capsule. Renal tubules were composed of cells with rounded vesicular nuclei. Hematopoietic tissue appeared normal in its content within the renal tissue, and melanomacrophages were also detected. In the Pyrogallol group, the kidney exhibited significant deterioration in renal tissue (Fig. 5b). There was thickening of Bowman's capsule around the Malpighian corpuscles, and the glomeruli showed vacuolar degeneration. Renal tubules had vacuolated cytoplasm and pyknotic nuclei, with necrotic areas also present. The hematopoietic tissue was excessively represented with densely packed components, and melanomacrophages were widespread throughout the renal tissue.

In the kidney sections from the pyrogallol + *S. platensis* group (Fig. 5c), mild improvement was observed compared to the pyrogallol group. Shrunken glomeruli of Malpighian corpuscles were observed, and renal tubular cells showed separation from their underlying cell membranes. Hematopoietic tissue was excessively present, and necrotic areas and melanomacrophages were detected.

In pyrogallol + *C. vulgaris* kidney sections (Fig. 5d), mild to moderate improvement in renal tissue was observed. Shrunken glomeruli of Malpighian corpuscles were observed, along with vacuolated renal tubular cells with pyknotic nuclei. Some necrotic areas and melanomacrophages were also present.

Moderate improvement was detected in the renal tissue of the pyrogallol + *M. oleifera* group (Fig. 5e). Normal Malpighian corpuscles and vesicular rounded nuclei of renal tubular cells were observed. Separation of some renal tubular cells from their basement membranes was detected, along with the presence of hematopoietic tissue and melanomacrophages.

Histopathological scoring for the above-mentioned lesions is represented in Table 3.

**Fig. 6:**
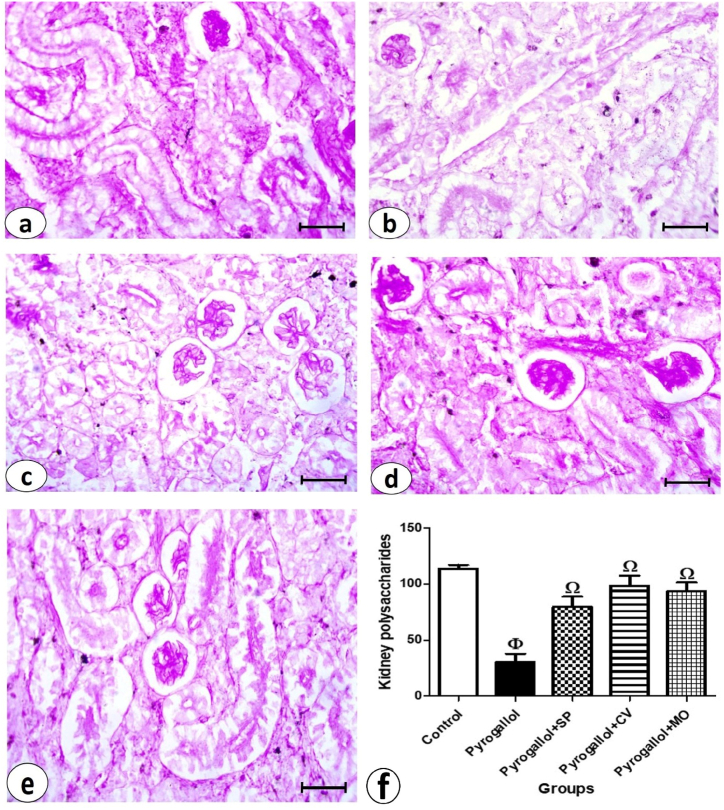
Examination of polysaccharides of kidney sections in *C. gariepinus*. **(a**–**e)**: photomicrographs of kidney sections stained by PAS stain (bars = 50 μm) showing: **(a)** control group with normal polysaccharides content as represented by magenta color in glomeruli, basement membranes and brush borders of renal tubules, **(b)** Pyrogallol group, **(c)** Pyrogallol + *S. platensis* (SP), **(d)** Pyrogallol + *C. vulgaris* (CV) and **(e)** Pyrogallol + *M. oleifera* (MO). **(f):** kidney polysaccharides of sections from all experimental groups. Bars represent means ± SE. Different letters indicate significant differences among treatments (p < 0.5).

Staining kidney sections with PAS staining revealed the polysaccharide content in the renal tissue (Fig. 6). The magenta color observed in the glomeruli of Bowman's capsule, basement membranes, and brush borders of renal tubules from the control group indicated a normal amount (Fig. 6a). A marked depletion of polysaccharide content was detected in the pyrogallol group (Fig. 6b), and this decrease was statistically significant compared to the control group (Fig. 6f). The number of polysaccharides increased significantly in the three treated groups (pyrogallol + *S. platensis*, pyrogallol + *C. vulgaris*, and pyrogallol + *M. oleifera*) compared to the pyrogallol group (Fig. 6c, d, e & f). There were no significant differences in polysaccharide content between the control group and the pyrogallol + *C. vulgaris* and pyrogallol + *M. oleifera* groups.

## 4 Discussion

As reported earlier [22], *C. gariepinus* encountered pyrogallol induced a marked drop in PCV [34] which could be due to hemodilution, osmoregulatory issues, or kidney function disruption [35]. The notable enhancement in PCV observed in our study, resulting from the dietary incorporation of microalgae, mirrors findings in *O. niloticus* supplemented with *C. vulgaris* after deltamethrin-induced hematotoxicity [36], as well as in *C. gariepinus* supplemented with *S. platensis* after chlorpyrifos exposure [37], and *O. niloticus* supplemented with *M. oleifera* after chlorpyrifos exposure [38].

The marked reduction in lymphocytic populations following pyrogallol exposure indicates weakness of immuno-potency, as documented earlier [22]. The decline in circulating lymphocytes could occur due to stress-triggered cell death in lymphocytes [39], heightened oxidative injury, and suppressed antioxidant shield [40]. The expansion of lymphocytic clones is crucial for phagocytosis and immune responses against xenobiotic agents [41] as seen in our microalgae-supplemented groups. The rise in lymphocytic count following feeding with microalgae-enriched diet confirms their immune-boosting impacts and stress-alleviating features of *C. vulgar*is (Dawood et al., 2020 [13], El-Son et al., 2022). The immune-related impacts of dietary *S. platensis* could be linked to its diverse bioactive components like phycocyanin, phycobilins, xanthophylls, and allophycocyanin (Nandeesha et al., 2001). The immune enhancement in *C. gariepinus* fed on the *M. oleifera*-enriched additive may be due to a variety of phytochemical ingredients (El-Son et al., 2022). These include the rich content of polyphenolic compounds, volatile oils, and vitamins known for their immune-boosting properties (Makkar et al., 2007). A previous study on the hematotoxic effects of pyrogallol in *C. gariepinus* triggered neutrophilia and eosinophilia [22]. On the contrary, the studied microalgae succeeded in counteracting these abnormalities by enhancing the expression of anti-inflammatory mediators and suppressing the expression of pro-inflammatory mediators [[42], [43], [44]].

The potential of pyrogallol to oxidize the sulfhydryl moiety located on fatty acid chains present within the cell membrane [22,45,46] might be the underlying reason for the poikilocytosis observed in this study. In addition, excessive generation of reactive oxygen species heightens lipid peroxidation, hampers intracellular protein function, and causes DNA harm. This process can impact mitochondrial oxidative phosphorylation, prompting the discharge of numerous inflammatory substances and ultimately resulting in execution of cell suicide [47,48]. The cytoprotective capability of microalgae aligns with findings observed in *O. niloticus* challenged with fipronil [49], largemouth bass (*Micropterus salmoides*) [44], and rats experiencing cobalt-induced apoptosis [50]. *S. platenesis* mitigates the genotoxic effects by alleviating the chromosomal structural aberrations and DNA mutations due to its exceptional polysaccharides, which boost DNA repairing enzymes [51]. Carotenoids, the active phytoconstituents in *S. platenesis*, encourage the activity of redox stabilizers and detoxifying enzymes, promoting cell proliferation [52]. *C. vulgar*is demonstrates an anti-apoptotic impact by boosting the expression of anti-apoptotic genes, while reducing the expression of pro-apoptotic genes in *M. salmoides* [44]. It increased the expression of peroxisome proliferator-activated receptor α, reduced activator protein 1 gene expression in the liver, and restored chromosomal stability and mitotic index of bone marrow cells in rats subjected to gibberellic acid-associated cytogenotoxicity [53]. Quercetin found in *M. oleifera* lessens micronucleus occurrences and chromosomal abnormalities by directly interacting with xanthine oxidase and nitric oxide synthase, thereby decreasing the oxidative burden inside the cellular milieu [54]. Kaempferol, a polyphenolic agent in *M. oleifera*, boosts b cell lymphoma-2 expression, consequently suppressing the presence of cell suicidal indicators like caspase-3 and poly (ADP-ribose) polymerase [55].

Given the dose and duration employed in our study, the oxidative infliction and inflammatory damage in our model might not have been intense enough to disrupt the cellular membranes or lead to the discharge of hepatic enzymes into the circulating blood. Further research is therefore needed to explore the dose-dependent effects of pyrogallol on liver function.

The elevated glucose levels noticed in pyrogallol-exposed fish might result from cortisol-induced hyperglycemia [45]. This observation is supported by the depletion of hepatic and renal glycogen reserves, evident in histochemical analysis, primarily attributed to increased mRNA expression of genes encoding enzymes of glycogenolysis [56]. The hypoglycemic activity of the studied microalgae is similar to that occurred in rainbow trout (*Oncorhynchus mykiss*) [57], crucian carp (*Carassius auratus*) [58], and *O. niloticus* [59]. The improvement in insulin sensitivity (Oriquat et al., 2019), glycogenesis, glycolysis (Hu et al., 2019), inhibition of gluconeogenesis, and activation of insulin receptors (El-Sakhawy et al., 2023) may contribute to the antihyperglycemic effect of *S. platensis*. *C. vulgar*is exhibits analogous action by reducing insulin resistance and safeguarding beta-cell function from peroxidative insult [60], as well as inhibiting pancreatic alpha-amylase [61]. Studies have demonstrated that *M. oleifera* can improve insulin resistance through multiple mechanisms. It activates the phosphoinositide 3-kinase/AKT and 5′ AMP-activated protein kinase pathways, enhances oxidative metabolism through the nicotinamide adenine dinucleotide-related deacetylase-peroxisome proliferator–activated receptor α pathway, and hinders fatty acid peroxidation [62,63].

The hypercholesterolemia following pyrogallol intoxication is aligned with [22]. The increase in TC levels might stem from alterations in liver cell permeability and disturbances in lipid metabolism, potentially associated with the buildup of the contaminants in the liver [64]. Consistent with other research, microalgae exhibited beneficial effects on lipid balance [[65], [66], [67]]. The hypolipidemic effects of *S. platensis* are thought to stem from its key components like linoleic acid, gamma linolenic acid, phycocyanin, phenolic compounds, and niacin [68]. *S. platensis* demonstrates its hypolipidemic effects by activating lecithin cholesterol acyltransferase, a crucial element in the reverse cholesterol transport process, and by hindering 3-hydroxy-3-methyl-glutaryl-coenzyme A reductase, an enzyme vital for the production of cholesterol [69]. *C. vulgar*is is an abundant provider of omega-3 polyunsaturated fatty acids which directly hinder acetyl-CoA carboxylases [70] and reduce the mRNA expression of sterol regulatory element–binding proteins [71], thereby decreasing the production and function of lipogenic enzymes. Lipid-lowering properties of *M. oleifera* might be linked to its chemical profile, containing alkaloids, flavonoids, saponins, and cardiac glycosides [72]. Saponins have the potential to lower cholesterol levels by either adhering to cholesterol in the intestines, thereby impeding its absorption, or by attaching to bile acids. This interaction may reduce the recycling of bile acids between the intestines and the liver, leading to their increased excretion in feces [73].

Although the oxidant and antioxidant biomarkers were measured in the serum, they reflect redox disturbances that may adversely impact the hepatic and renal structures [[74], [75], [76]]. The redox alterations induced by pyrogallol, marked by increased serum MDA levels and decreased serum TAC and SOD activity, align with the findings of Hamed et al. [77,78]). Like other chemotoxicants, this can be explained by the overproduction of reactive oxygen species [10], along with the downregulation of antioxidant gene expression [79], redox-linked transcription factors, and antioxidant responsive mediators [80]. The oxidative stress caused by pyrogallol intoxication may be associated with the histological lesions and cytological modifications [81]. The ability of microalgae to counteract the redox abnormalities induced by pyrogallol is comparable to the effects observed in *O. niloticus* exposed to imidacloprid [82], deltamethrin [36], and oxyfluorfen toxicity. The presence of bioactive components namely β-carotene, C-phycocyanin [83], γ-linolenic acid and α-tocopherol [84] could underlie the marked antioxidant ability of *S. platenesis* against pyrogallol-induced oxidative injuries. These compounds, whether independently or synergistically, neutralize free radicals like, alkoxyl, peroxyl, and hydroxyl radicals [85]. Additionally, C-phycocyanin can decrease nitrite generation and block lipid peroxidation [86]. The defensive characteristics of *C. vulgaris* against the disruption in antioxidant shield may be related to its carotenoid profile, including β-carotene, lutein, canthaxanthin, and astaxanthin [87]. They are effective in quenching the singlet oxygen and eliminating reactive oxidants [88]. The phenolic constituents in *M. oleifera*, including kaempferol and quercetin glucosides [89], along with its total flavonoid content [90], are responsible for its antioxidant activity.

Increased serum IL-6 levels, inflammatory cell infiltration in liver tissue, along with an elevated count of blood neutrophils and eosinophils, suggest that inflammation may play a significant role in pyrogallol toxicity. In a previous study [76], pro-inflammatory cytokines, including IL-1 beta and IL-6, were found to be elevated in the serum of *C. gariepinus* following pyrogallol contamination. This outcome could be attributed to the stimulation of the inflammatory cascade through the upregulation of the NF-κB signaling pathway [91]. Pyrogallol depletes dissolved oxygen levels in water tanks [22]. The depletion of oxygen in the ecosystem has been associated with increased levels of pro-inflammatory mediators in Chilean salmon (*Oncorhynchus kisutch*) [92]. Similar to other reports [46], inflammatory cell accumulation was observed in the intestinal and muscular tissues of pyrogallol-intoxicated *C. gariepinus.* This finding might be due to the release of chemoattractants, which act as mediators for inflammatory cellular recruitment. IL-6 plays a role in neutrophil migration by inducing the production of chemokines, such as IL-8 and monocyte chemoattractant protein-1, and by triggering the expression of adhesion molecules on endothelial cells [93]. Following the trafficking of neutrophils to the challenge site, they release chemokines to stimulate tissue-resident cells and recruit more leukocytes to the injured area, thereby intensifying inflammation [94]. Inflammatory response can lead to a marked increase in eosinophil count [95]. Eosinophils release inflammatory mediators through degranulation [96] and function as antigen-presenting cells during inflammation (Jung et al., 2008). A previous study on the hematotoxic effects of pyrogallol in *C. gariepinus* reported neutrophilia and eosinophilia [22]. Conversely, the studied microalgae succeeded in counteracting these abnormalities by enhancing the expression of anti-inflammatory mediators and suppressing the expression of pro-inflammatory mediators [42,43].

Free radicals have the potential to harm the renal cells directly, activate intracellular signaling routes, or prompt systemic reactions that result in kidney damage [97]. In a previous cited article, pyrogallol-treated *C. gariepinus* displayed extensive damage to the tubular structure, including complete splitting of the tubular epithelium with the loss of the basement membrane and various changes in cell nuclei. Additionally, the intertubular hematopoietic tissues exhibited higher content of RBCs alongside increased pigmented cell counts [46,76]. In parallel with the histopathological disruptions in renal tissues and in accordance with previous research [22], serum creatinine levels markedly raised in the pyrogallol group. Estimation of creatinine can be applied to identify renal filtration rate and as an evaluation for renal inadequacy [98]. The increased creatinine can be ascribed to the reducing effect of pyrogallol on glomerular filtration or increase in protein breakdown rates [99].

Xenobiotics are recognized for causing liver damage through generating metabolic intermediates that can interact with cellular elements resulting in oxidative injury, antioxidants exhaustion, lipid peroxidation, and heightened membrane penetrability [100,101]. The cellular alterations in the liver of pyrogallol-exposed *C. gariepinus* mirror those documented previously [46], and these changes are linked to the capacity of pyrogallol to stimulate pro-inflammatory cytokines which play important role in hepatic parenchymal cell damage [102]. At the cellular level, cytoplasmic vacuoles are formed from elements of the endoplasmic reticulum or endosomal-lysosomal organelles due to cellular osmotic disturbance and exhaustion of ATP reserve [103]. The histopathological deteriorations observed in the kidney of fish exposed to pyrogallol resemble those reported in a previous study [76]. Extensive necrosis in hepatic tissue and the degeneration of renal tubules and glomerular tuft are commonly documented during exposure to various toxicants [13,43,104] and may be mediated by the disruption in cytoskeletal elements [105]. Pyknosis, the irreversible condensation of chromatin within a cell's nucleus, serves as an indicator of cellular apoptosis [106]. An elevated occurrence of melanomacrophage aggregates in the kidney signifies notable tissue lesions and elevated immune response [107]. The observed histological enhancements from the investigated microalgae are consistent with findings previously documented in studies by Refs. [37,108,109]. The reduction in quantities of MMCs in microalgae-supplemented groups could be due to their immunostimulatory properties [110,111]. The hematopoietic system in fish exhibits significant homeostatic potential, typically compensating for cell loss through the initiation of mitotic replication [112]. The cytoprotective effect of *S. platensis* arises from its ability to up-regulate the expression of proliferating cell nuclear antigen which is responsible for DNA replication and regulation of cell cycle [113]. In *C. gariepinus*, *C. vulgar*is counteracted the hepatorenal toxicity induced by microplastic particles by restoring the carbohydrate content in the microvilli and underlying membranes of kidney tubules and glomeruli, as well as within the cytoplasm of liver cells [114]. *C. vulgar*is possesses chlorella growth mediators, promoting cellular multiplication and tissue regeneration. It also activates the immunity to assist in clearing away deceased cells [115]. *M. oleifera* prompted the expression of PCNA and Ki-67 in the renal tissues of rats experiencing melamine-induced nephrotoxicity, indicating an improved capacity for cellular multiplication and renewal [116].

## 5 Conclusions

Based on our results, exposure of *C. gariepinus* to pyrogallol led to alterations in blood granulocytes percent, erythrocytic cellular and nuclear features, and the redox defensive mechanism. Pyrogallol did not induce hepato-renal dysfunction and microalgae intervention did not enhance hepato-renal function. Based on the dose and duration used in our study, the oxidative burden and inflammatory effects in our experimental model may not have reached a threshold sufficient to disrupt the cellular membrane and cause the leakage of hepatic metabolizing enzymes into the bloodstream. Nevertheless, pyrogallol caused elevated serum glucose, TC, and creatinine levels, indicating metabolic deterioration confirmed by cytopathological lesions. Conversely, the investigated microalgae showed promise as potential remedies for mitigating these abnormalities. In this regard, *M. oleifera* was the most effective, followed by *C. vulgaris*, with *S. platensis* being the least effective. Consequently, incorporating these nutritional supplements into the diet could be pivotal in the aquaculture sector to alleviate the impact of environmental pollutants. However, dose-dependent studies are strongly recommended to thoroughly investigate the toxicological effects of pyrogallol on hepato-renal function and the potential benefits of microalgae.

## CRediT authorship contribution statement

**Mohamed Hamed:** Writing – review & editing, Writing – original draft, Methodology, Investigation, Conceptualization. **Nasser S. Abou Khalil:** Writing – review & editing, Writing – original draft, Methodology, Investigation. **Alshaimaa A.I. Alghriany:** Writing – review & editing, Writing – original draft, Funding acquisition, Formal analysis. **Alaa El-Din H. Sayed:** Writing – review & editing, Writing – original draft, Methodology, Investigation, Conceptualization.

## Data availability statement

All relevant raw data will be freely available from the authors.

## Funding statement

This research received no external funding.

## Declaration of competing interest

The authors declare that they have no known competing financial interests or personal relationships that could have appeared to influence the work reported in this paper.

## References

[1] Lal J., Singh S.K., Biswas P., Debbarma R., Rather M.A., Amin A., Hajam Y.A., Jamwal A., Ahmad I. (2023). Xenobiotics in Aquatic Animals: Reproductive and Developmental Impacts.

[2] El-Houseiny W., Anter R.G.A., Arisha A.H., Mansour A.T., Safhi F.A., Alwutayd K.M., Elshopakey G.E., Abd El-Hakim Y.M., Mohamed E.M.M. (2023). Growth retardation, oxidative stress, immunosuppression, and inflammatory disturbances induced by herbicide exposure of catfish. Clarias gariepinus, and the Alleviation Effect of Dietary Wormwood, Artemisia cina.

[3] Gokul T., Kumar K.R., Prema P., Arun A., Balaji P., Faggio C. (2023). Particulate pollution and its toxicity to fish: an overview. Comp. Biochem. Physiol. C Toxicol. Pharmacol..

[4] Novak A.J., Trauner D. (2022). Pages 1-46 Progress in the Chemistry of Organic Natural Products 118.

[5] Kim Y.-J., Kim H.-Y., Lee J.-D., Kim H.-Y., Im J.-E., Kim K.-B. (2022).

[6] Rocchetti G., Blasi F., Montesano D., Ghisoni S., Marcotullio M.C., Sabatini S., Cossignani L., Lucini L. (2019). Impact of conventional/non-conventional extraction methods on the untargeted phenolic profile of Moringa oleifera leaves. Food Res. Int..

[7] Xiong S.-L., Lim G.T., Yin S.-J., Lee J., Si Y.-X., Yang J.-M., Park Y.-D., Qian G.-Y. (2019). The inhibitory effect of pyrogallol on tyrosinase activity and structure: integration study of inhibition kinetics with molecular dynamics simulation. Int. J. Biol. Macromol..

[8] Upadhyay G., Gupta S.P., Prakash O., Singh M.P. (2010). Pyrogallol-mediated toxicity and natural antioxidants: triumphs and pitfalls of preclinical findings and their translational limitations. Chem. Biol. Interact..

[9] Abirami G., Alexpandi R., Sudhin S., Durgadevi R., Roshni P.S., Kumar P., Veera Ravi A. (2023). Pyrogallol downregulates the expression of virulence-associated proteins in Acinetobacter baumannii and showing anti-infection activity by improving non-specific immune response in zebrafish model. Int. J. Biol. Macromol..

[10] Baruah K., Duy Phong H.P., Norouzitallab P., Defoirdt T., Bossier P. (2015). The gnotobiotic brine shrimp (Artemia franciscana) model system reveals that the phenolic compound pyrogallol protects against infection through its prooxidant activity. Free Radic. Biol. Med..

[11] Hamed M., Soliman H.A.M., Sayed A.E.-D.H. (2019). Ameliorative effect of Spirulina platensis against lead nitrate–induced cytotoxicity and genotoxicity in catfish Clarias gariepinus. Environ. Sci. Pollut. Control Ser..

[12] Ismail R.F., Saleh N.E., Sayed A.E.D.H. (2021). Impacts of microplastics on reproductive performance of male tilapia (Oreochromis niloticus) pre-fed on Amphora coffeaeformis. Environ. Sci. Pollut. Control Ser..

[13] Abdel-Latif H.M., Soliman A.A., Khaled A.A., Kord M., Abdel-Tawwab M., Darwish S., Grana Y.S., Zaki M., Nour A.-E., Ali E. (2022). Growth performance, antioxidant activities, and immunological responses of hapa-reared thinlip mullet (Liza ramada) juveniles fed on diets supplemented with spirulina (Arthrospira platensis). Fish Shellfish Immunol..

[14] El-Din H Sayed A., Hamed M., Ismail R.F. (2022). Natural antioxidants can improve microplastics-induced male reproductive impairment in the african catfish (Clarias gariepinus). Front. Environ. Sci..

[15] Mohamed I.A., Soliman H.A.M., Hana M., Lee J.S., Sayed A.E.D.H. (2023). Toxicity of mixture of polyethylene microplastics and up Grade® pesticide on Oreochromis niloticus juvenile: I. Hemato-biochemical and histopathological alterations. Environ. Toxicol. Pharmacol..

[16] Mawed S.A., Centoducati G., Farag M.R., Alagawany M., Abou-Zeid S.M., Elhady W.M., El-Saadony M.T., Di Cerbo A., Al-Zahaby S.A. (2022). Dunaliella salina microalga restores the metabolic equilibrium and ameliorates the hepatic inflammatory response induced by zinc oxide nanoparticles (ZnO-NPs) in male zebrafish. Biology.

[17] Abdelhamid F.M., Elshopakey G.E., Aziza A.E. (2020). Ameliorative effects of dietary Chlorella vulgaris and β-glucan against diazinon-induced toxicity in Nile tilapia (Oreochromis niloticus). Fish Shellfish Immunol..

[18] Ibrahim R.E., Ghamry H.I., Althobaiti S.A., Almalki D.A., Shakweer M.S., Hassan M.A., Khamis T., Abdel-Ghany H.M., Ahmed S.A.A. (2023). Moringa oleifera and Azadirachta indica leaves enriched diets mitigate chronic oxyfluorfen toxicity induced immunosuppression through disruption of pro/anti-inflammatory gene pathways, alteration of antioxidant gene expression, and histopathological alteration in Oreochromis niloticus.

[19] Mohamed I.A., Hamed M., Abdel-Tawab H.S., Mansour S., Soliman H.A.M., Lee J.S., El-Din H.S.A. (2022). Multi-biomarkers approach to assess the toxicity of novel insecticide (Voliam flexi®) on Clarias gariepinus: from behavior to immunotoxicity. Fish Shellfish Immunol..

[20] Javed M., Usmani N. (2015). Impact of heavy metal toxicity on hematology and glycogen status of fish: a review. Proc. Natl. Acad. Sci. India B Biol. Sci..

[21] Wolf J.C., Wheeler J.R. (2018). A critical review of histopathological findings associated with endocrine and non-endocrine hepatic toxicity in fish models. Aquat. Toxicol..

[22] Hamed M., Martyniuk C.J., Said R.E., Soliman H.A., Badrey A.E., Hassan E.A., Abdelhamid H.N., Osman A.G., Sayed A.E.-D.H. (2023). Exposure to pyrogallol impacts the hemato-biochemical endpoints in catfish (Clarias gariepinus). Environmental Pollution.

[23] Sayed A.E.-D.H., Hamed M., Soliman H.A.M., Authman M.M.N. (2022). The protective role of lycopene against toxic effects induced by the herbicide Harness® and its active ingredient acetochlor on the African catfish Clarias gariepinus (Burchell, 1822). Environ. Sci. Pollut. Control Ser..

[24] Sayed A.E.-D.H., AbdAllah E.A., Hamed M., Soliman H.A.M. (2020). Hepato-nephrotoxicity in late juvenile of Oreochromis niloticus exposed to gibberellic acid: ameliorative effect of Spirulina platensis. Pestic. Biochem. Physiol..

[25] Sayed A.E.-D.H., Hamed M., Ismail R.F. (2022). Natural antioxidants can improve microplastics-induced male reproductive impairment in the african catfish (Clarias gariepinus). Front. Environ. Sci..

[26] El-Kassas S., Aljahdali N., Abdo S.E., Alaryani F.S., Moustafa E.M., Mohamed R., Abosheashaa W., Abdulraouf E., Helal M.A., Shafi M.E., El-Saadony M.T., El-Naggar K., Conte-Junior C.A. (2022). Moringa oleifera leaf powder dietary inclusion differentially modulates the antioxidant, inflammatory, and histopathological responses of normal and aeromonas hydrophila -infected mono-sex nile Tilapia (Oreochromis niloticus). Page 918933 Frontiers in veterinary science.

[27] Kaya H., Çelik E.Ş., Yılmaz S., Tulgar A., Akbulut M., Demir N. (2015). Hematological, serum biochemical, and immunological responses in common carp (Cyprinus carpio) exposed to phosalone. Comp. Clin. Pathol..

[28] Sayed A.E.D.H., Kataoka C., Oda S., Kashiwada S., Mitani H. (2018). Sensitivity of medaka (Oryzias latipes) to 4-nonylphenol subacute exposure; erythrocyte alterations and apoptosis. Environ. Toxicol. Pharmacol..

[29] Bricknell I., Bowden T., Bruno D., MacLachlan P., Johnstone R., Ellis A. (1999). Susceptibility of Atlantic halibut, Hippoglossus hippoglossus (L.) to infection with typical and atypical Aeromonas salmonicida. Aquaculture.

[30] Rifai N. (2023).

[31] Ohkawa H., Ohishi N., Yagi K. (1979). Assay for lipid peroxides in animal tissues by thiobarbituric acid reaction. Anal. Biochem..

[32] Nishikimi M., Appaji N., Yagi K. (1972). The occurrence of superoxide anion in the reaction of reduced phenazine methosulfate and molecular oxygen. Biochem. Biophys. Res. Commun..

[33] Hanington P.C., Belosevic M. (2007). Interleukin-6 family cytokine M17 induces differentiation and nitric oxide response of goldfish (Carassius auratus L.) macrophages. Dev. Comp. Immunol..

[34] Iheanacho S.C., Odo G.E. (2020). Neurotoxicity, oxidative stress biomarkers and haematological responses in African catfish (Clarias gariepinus) exposed to polyvinyl chloride microparticles. Comp. Biochem. Physiol. C Toxicol. Pharmacol..

[35] Abdel-Tawwab M., Wafeek M. (2017). Fluctuations in water temperature affected waterborne cadmium toxicity: hematology, anaerobic glucose pathway, and oxidative stress status of Nile tilapia, Oreochromis niloticus (L.). Aquaculture.

[36] Mahmoud E.A., El-Sayed B.M., Mahsoub Y.H., Neamat-Allah A.N. (2020). Effect of Chlorella vulgaris enriched diet on growth performance, hemato-immunological responses, antioxidant and transcriptomics profile disorders caused by deltamethrin toxicity in Nile tilapia (Oreochromis niloticus). Fish Shellfish Immunol..

[37] Mokhbatly A.-A.A., Assar D.H., Ghazy E.W., Elbialy Z., Rizk S.A., Omar A.A., Gaafar A.Y., Dawood M.A. (2020). The protective role of spirulina and β-glucan in African catfish (Clarias gariepinus) against chronic toxicity of chlorpyrifos: hemato-biochemistry, histopathology, and oxidative stress traits. Environ. Sci. Pollut. Control Ser..

[38] Ibrahim R.E., El-Houseiny W., Behairy A., Mansour M.F., Abd-Elhakim Y.M. (2019). Ameliorative effects of Moringa oleifera seeds and leaves on chlorpyrifos-induced growth retardation, immune suppression, oxidative stress, and DNA damage in Oreochromis niloticus. Aquaculture.

[39] Davis A., Maney D., Maerz J. (2008). The use of leukocyte profiles to measure stress in vertebrates: a review for ecologists. Funct. Ecol..

[40] Chen L., Diao J., Zhang W., Zhang L., Wang Z., Li Y., Deng Y., Zhou Z. (2019). Effects of beta-cypermethrin and myclobutanil on some enzymes and changes of biomarkers between internal tissues and saliva in reptiles (Eremias argus). Chemosphere.

[41] Reynaud S., Deschaux P. (2005). The effects of 3-methylcholanthrene on lymphocyte proliferation in the common carp (Cyprinus carpio L.). Toxicology.

[42] Khalil S.R., Reda R.M., Awad A. (2017). Efficacy of Spirulina platensis diet supplements on disease resistance and immune-related gene expression in Cyprinus carpio L. exposed to herbicide atrazine. Fish Shellfish Immunol..

[43] Ibrahim R.E., Ghamry H.I., Althobaiti S.A., Almalki D.A., Shakweer M.S., Hassan M.A., Khamis T., Abdel-Ghany H.M., Ahmed S.A. (2022). Moringa oleifera and Azadirachta indica Leaves enriched diets mitigate chronic oxyfluorfen toxicity induced immunosuppression through disruption of pro/anti-inflammatory gene pathways, alteration of antioxidant gene expression, and histopathological Alteration in Oreochromis niloticus. Fishes.

[44] Yu H., Liang H., Ge X., Zhu J., Wang Y., Ren M., Chen X. (2022). Dietary chlorella (Chlorella vulgaris) supplementation effectively improves body color, alleviates muscle inflammation and inhibits apoptosis in largemouth bass (Micropterus salmoides). Fish Shellfish Immunol..

[45] Abdel-Tawwab M., Monier M.N., Hoseinifar S.H., Faggio C. (2019). Fish response to hypoxia stress: growth, physiological, and immunological biomarkers. Fish Physiol. Biochem..

[46] Hamed M., Said R.E.M., Soliman H.A.M., Osman A.G.M., Martyniuk C.J. (2024). Immunotoxicological, histopathological, and ultrastructural effects of waterborne pyrogallol exposure on African catfish (Clarias gariepinus). Chemosphere.

[47] Dobi A., Bravo S.B., Veeren B., Paradela-Dobarro B., Álvarez E., Meilhac O., Viranaicken W., Baret P., Devin A., Rondeau P. (2019). Advanced glycation end-products disrupt human endothelial cells redox homeostasis: new insights into reactive oxygen species production. Free Radic. Res..

[48] Angelova P.R., Dinkova-Kostova A.T., Abramov A.Y. (2021). Assessment of ROS production in the mitochondria of live cells. Reactive Oxygen Species: Methods and Protocols.

[49] Fadl S.E., Elbialy Z.I., Abdo W., Saad A.H., Aboubakr M., Abdeen A., Elkamshishi M.M., Salah A.S., El-Mleeh A., Almeer R., Aleya L., Abdel-Daim M.M., Najda A., Abdelhiee E.Y. (2022). Ameliorative effect of Spirulina and Saccharomyces cerevisiae against fipronil toxicity in Oreochromis niloticus. Ecotoxicol. Environ. Saf..

[50] Khalil S.R., El Bohi K.M., Khater S., Abd El-Fattah A.H., Mahmoud F.A., Farag M.R. (2020). Moringa oleifera leaves ethanolic extract influences DNA damage signaling pathways to protect liver tissue from cobalt -triggered apoptosis in rats. Ecotoxicol. Environ. Saf..

[51] Aly F.M., Kotb A.M., Hammad S. (2018). Effects of Spirulina platensis on DNA damage and chromosomal aberration against cadmium chloride-induced genotoxicity in rats. Environ. Sci. Pollut. Control Ser..

[52] De Mejia E.G., Zhang Q., Penta K., Eroglu A., Lila M.A. (2020). The colors of health: chemistry, bioactivity, and market demand for colorful foods and natural food sources of colorants. Annu. Rev. Food Sci. Technol..

[53] Khadrawy S.M., Mohamed D.S., Hassan R.M., Abdelgawad M.A., Ghoneim M.M., Alshehri S., Shaban N.S. (2023). Royal jelly and chlorella vulgaris mitigate gibberellic acid-induced cytogenotoxicity and hepatotoxicity in rats via modulation of the pparα/AP-1 signaling pathway and suppression of oxidative stress and inflammation. Foods.

[54] Onur M., Yalçın E., Çavuşoğlu K., Acar A. (2023). Elucidating the toxicity mechanism of AFM2 and the protective role of quercetin in albino mice. Sci. Rep..

[55] Rajendran P., Ammar R.B., Al-Saeedi F.J., Mohamed M.E., ElNaggar M.A., Al-Ramadan S.Y., Bekhet G.M., Soliman A.M. (2020). Kaempferol inhibits zearalenone-induced oxidative stress and apoptosis via the PI3K/Akt-mediated Nrf2 signaling pathway: in vitro and in vivo studies. Int. J. Mol. Sci..

[56] Li M., Wang X., Qi C., Li E., Du Z., Qin J.G., Chen L. (2018). Metabolic response of Nile tilapia (Oreochromis niloticus) to acute and chronic hypoxia stress. Aquaculture.

[57] Yeganeh S., Teimouri M., Amirkolaie A.K. (2015). Dietary effects of Spirulina platensis on hematological and serum biochemical parameters of rainbow trout (Oncorhynchus mykiss). Res. Vet. Sci..

[58] Shi X., Luo Z., Chen F., Wei C.-C., Wu K., Zhu X.-M., Liu X. (2017).

[59] Gbadamosi O.K., Fasakin A.E., Adebayo O.T. (2016). Hepatoprotective and stress-reducing effects of dietary Moringa oleifera extract against Aeromonas hydrophila infections and transportation-induced stress in Nile tilapia. Oreochromis niloticus (Linnaeus 1757) fingerlings.

[60] Abdella A., Abou-Elazm F.I., El-Far S.W. (2023). Pharmacological effects of lactobacillus casei ATCC 7469 fermented soybean and green microalgae, chlorella vulgaris, on diabetic rats. Microbiol. Res..

[61] Sahoo S., Samantaray M., Jena M., Gosu V., Bhuyan P.P., Shin D., Pradhan B. (2023). In Vitro and in silico studies to explore potent antidiabetic inhibitor against human pancreatic alpha-amylase from the methanolic extract of the green microalga Chlorella vulgaris. J. Biomol. Struct. Dyn..

[62] Bao Y., Xiao J., Weng Z., Lu X., Shen X., Wang F. (2020). A phenolic glycoside from Moringa oleifera Lam. improves the carbohydrate and lipid metabolisms through AMPK in db/db mice. Food Chem..

[63] Duranti G., Maldini M., Crognale D., Sabatini S., Corana F., Horner K., Ceci R. (2021). Moringa oleifera leaf extract influences oxidative metabolism in C2C12 myotubes through SIRT1-PPARα pathway. Phytomedicine.

[64] Yousef M.I., El-Demerdash F., Kamel K., Al-Salhen K. (2003). Changes in some hematological and biochemical indices of rabbits induced by isoflavones and cypermethrin. Toxicology.

[65] Carneiro W.F., Castro T.F.D., Orlando T.M., Meurer F., Paula D.A.d.J., Virote B.d.C.R., Vianna A.R.B., Murgas L.D.S.J.A. (2020).

[66] El‐Kassas S., Abdo S.E., Abosheashaa W., Mohamed R.A., Moustafa E.M., Helal M.A., El-Naggar K.J.A.R. (2020).

[67] Mohammadiazarm H., Maniat M., Ghorbani-Jezeh K., Ghotbeddin N.J.A.N. (2020). Blood Indices and Total Pigmentation.

[68] Al-Dhabi N.A., Valan Arasu M. (2016). Quantification of phytochemicals from commercial Spirulina products and their antioxidant activities. Evid. base Compl. Alternative Med..

[69] Ama Moor V.J., Nya Biapa P.C., Nono Njinkio B.L., Moukette Moukette B., Sando Z., Kenfack C., Ateba B., Ngo Matip M.E., Pieme C.A., Ngogang J. (2017). Hypolipidemic effect and activation of Lecithin Cholesterol Acyl Transferase (LCAT) by aqueous extract of Spirulina platensis during toxicological investigation. BMC nutrition.

[70] Jacobson T.A. (2008). Role of n− 3 fatty acids in the treatment of hypertriglyceridemia and cardiovascular disease. The American journal of clinical nutrition.

[71] Moon Y.-A., Hammer R.E., Horton J.D. (2009). Deletion of ELOVL5 leads to fatty liver through activation of SREBP-1c in mice. Journal of lipid research.

[72] Oyedepo T., Babarinde S., Ajayeoba T. (2013). Evaluation of anti-hyperlipidemic effect of aqueous leaves extract of Moringa oleifera in alloxan induced diabetic rats. Int. J. Biochem. Res. Rev..

[73] Rotimi S.O., Omotosho O.E., Rotimi O.A. (2011). Persistence of acidosis in alloxan-induced diabetic rats treated with the juice of Asystasia gangetica leaves. Phcog. Mag..

[74] Naiel M.A., Ismael N.E., Shehata S.A. (2019). Ameliorative effect of diets supplemented with rosemary (Rosmarinus officinalis) on aflatoxin B1 toxicity in terms of the performance, liver histopathology, immunity and antioxidant activity of Nile Tilapia (Oreochromis niloticus). Aquaculture.

[75] Rahman A.N.A., ElHady M., Hassanin M.E., Mohamed A.A.-R. (2019). Alleviative effects of dietary Indian lotus leaves on heavy metals-induced hepato-renal toxicity, oxidative stress, and histopathological alterations in Nile tilapia, Oreochromis niloticus (L.). Aquaculture.

[76] Hamed M., Soliman H.A., Said R.E., Martyniuk C.J., Osman A.G., Sayed A.E.-D.H. (2024). Oxidative stress, antioxidant defense responses, and histopathology: biomarkers for monitoring exposure to pyrogallol in Clarias gariepinus. J. Environ. Manag..

[77] Hamed M., Martyniuk C.J., Soliman H.A.M., Osman A.G.M., Said R.E.M. (2024). Neurotoxic and cardiotoxic effects of pyrogallol on catfish (Clarias gariepinus). Environ. Toxicol. Pharmacol..

[78] Hamed M., Said R.E.M., Martyniuk C.J., Soliman H.A.M., Sayed A.E.-D.H., Osman A.G.M. (2024). Reproductive and endocrine-disrupting toxicity of pyrogallol in catfish (Clarias gariepinus). Environmental Pollution.

[79] Mansour W.A.A., Abdelsalam N.R., Tanekhy M., Khaled A.A., Mansour A.T. J.C.b., Toxicology p., Cbp p. (2021).

[80] Jiang W.d., Liu Y., Jiang J., Wu P., Feng L., Zhou X.q.J.A.t. (2015).

[81] Zhou Y.-C., Gui L., Wei W., Xu E.G., Zhou W., Sokolova I.M., Li M., Wang Y.J. A.t. (2023).

[82] Abdel‐Tawwab M., El-saadawy H.A., El-Belbasi H.I., Abd El-hameed S.A.A., Attia A.A. J.C.b., Toxicology p., Cbp p. (2021). Antioxidant, and Immune Responses of Nile tilapia.

[83] Wu Q., Liu L., Miron A., Klimova B.F., Wan D., Kuča K.J. A.o.T. (2016).

[84] Yaakob Z, Ali E, Zainal A, Mohamad M, Takriff MS (2014 May 19). An overview: biomolecules from microalgae for animal feed and aquaculture. J Biol Res (Thessalon).

[85] Wiley J., Sons L., Upasani K.C.D., Balaraman R.J.P.R. (2003).

[86] Romay C., González R., Ledón N., Remirez D.C., Rimbau V.J. C.p., science p. (2003). C-phycocyanin: a biliprotein with antioxidant, anti-inflammatory and neuroprotective effects.

[87] Goiris K., Muylaert K., Fraeye I., Foubert I., De Brabanter J., De Cooman L.J. (2012).

[88] Tamura H., Ishikita H.J. (2020).

[89] Llorent-Martínez E.J., Gordo-Moreno A.I., Fernández-de Córdova M.L., Ruiz-Medina A.J.A. (2023).

[90] Vats S., Gupta T.J.P., Plants M.B.o. (2017). Evaluation of bioactive compounds and antioxidant potential of hydroethanolic extract of Moringa oleifera Lam. from Rajasthan, India.

[91] Ekozin A., Otuechere C.A., Adewuyi A. (2022). Apocynin loaded silver nanoparticles displays potent in vitro biological activities and mitigates pyrogallol-induced hepatotoxicity. Chem. Biol. Interact..

[92] Martínez D., De Lázaro O., Cortés P., Oyarzún-Salazar R., Paschke K., Vargas-Chacoff L. (2020). Hypoxia modulates the transcriptional immunological response in Oncorhynchus kisutch. Fish Shellfish Immunol..

[93] Suzuki M., Hashizume M., Yoshida H., Mihara M. (2010). Anti-inflammatory mechanism of tocilizumab, a humanized anti-IL-6R antibody: effect on the expression of chemokine and adhesion molecule. Rheumatol. Int..

[94] Sommer F., Torraca V., Meijer A.H. (2020). Chemokine receptors and phagocyte biology in zebrafish. Front. Immunol..

[95] Valent P. (2009). Pathogenesis, classification, and therapy of eosinophilia and eosinophil disorders. Blood Rev..

[96] Miike S., Kita H. (2003). Human eosinophils are activated by cysteine proteases and release inflammatory mediators. Journal of allergy and clinical immunology.

[97] Yaribeygi H., Farrokhi F.R., Rezaee R., Sahebkar A. (2018). Oxidative stress induces renal failure: a review of possible molecular pathways. J. Cell. Biochem..

[98] Sebaei M.E., Shathele M., El-Bahr S.M. (2022). Impaired cytokines gene expression by diazinon and its relation with blood chemistry and oxidative stress biomarkers in Nile Tilapia (Oreochromis niloticus). The Thai Journal of Veterinary Medicine.

[99] Akturk O., Demirin H., Sutcu R., Yilmaz N., Koylu H., Altuntas I. (2006). The effects of diazinon on lipid peroxidation and antioxidant enzymes in rat heart and ameliorating role of vitamin E and vitamin C. Cell biology and toxicology.

[100] Holt M.P., Ju C. (2006). Mechanisms of drug-induced liver injury. AAPS J..

[101] Grattagliano I., Bonfrate L., Diogo C.V., Wang H.H., Wang D.Q., Portincasa P. (2009). Biochemical mechanisms in drug-induced liver injury: certainties and doubts. World J. Gastroenterol.: WJG.

[102] Fontes-Cal T.C., Mattos R.T., Medeiros N.I., Pinto B.F., Belchior-Bezerra M., Roque-Souza B., Dutra W.O., Ferrari T.C., Vidigal P.V., Faria L.C. (2021). Crosstalk between plasma cytokines, inflammation, and liver damage as a new strategy to monitoring NAFLD progression. Front. Immunol..

[103] Shubin A.V., Demidyuk I.V., Komissarov A.A., Rafieva L.M., Kostrov S.V.J.O. (2016).

[104] Abdel-Tawwab M., Khalil R.H., Younis N.A., Abo Selema T.A., Saad A.H., El-Werwary S.O., Gouda A.H., Soliman A.M., Shady S.H., Monier M.N. (2023). Saccharomyces cerevisiae supplemented diets mitigate the effects of waterborne cadmium toxicity on gilthead seabream (Sparus aurata L.): growth performance, haemato-biochemical, stress biomarkers, and histopathological investigations. Vet. Res. Commun..

[105] Zhou M., Tu W.-w., Xu J. (2015). Mechanisms of microcystin-LR-induced cytoskeletal disruption in animal cells. Toxicon.

[106] Leyva-Soto A., Chavez-Santoscoy R.A., Porras O., Hidalgo-Ledesma M., Serrano-Medina A., Ramírez-Rodríguez A.A., Castillo-Martinez N.A. (2021). Epicatechin and quercetin exhibit in vitro antioxidant effect, improve biochemical parameters related to metabolic syndrome, and decrease cellular genotoxicity in humans. Food Res. Int..

[107] Sayed A.H., Younes H.A.M. (2016). Melanomacrophage centers in Clarias gariepinus as an immunological biomarker for toxicity of silver nanoparticles. Journal of Microscopy Ultrastructure.

[108] Zahran E., Awadin W., Risha E., Khaled A.A., Wang T. (2019). Dietary supplementation of Chlorella vulgaris ameliorates chronic sodium arsenite toxicity in Nile tilapia Oreochromis niloticus as revealed by histopathological, biochemical and immune gene expression analysis. Fisheries science.

[109] Abdelhiee E.Y., Elbialy Z.I., Saad A.H., Dawood M.A., Aboubakr M., El-Nagar S.H., El-Diasty E.M., Salah A.S., Saad H.M., Fadl S.E. (2021). The impact of Moringa oleifera on the health status of Nile tilapia exposed to aflatoxicosis. Aquaculture.

[110] Mahmoud M.M., El-Lamie M.M., Kilany O.E., Dessouki A.A. (2018). Spirulina (Arthrospira platensis) supplementation improves growth performance, feed utilization, immune response, and relieves oxidative stress in Nile tilapia (Oreochromis niloticus) challenged with Pseudomonas fluorescens. Fish Shellfish Immunol..

[111] Sayed A.E.-D.H., Hamed M., Soliman H.A.M. (2021). Spirulina platensis alleviated the hemotoxicity, oxidative damage and histopathological alterations of hydroxychloroquine in catfish (Clarias gariepinus). Front. Physiol..

[112] Witeska M., Kondera E., Bojarski B. (2023). Hematological and hematopoietic analysis in fish toxicology—a review. Animals.

[113] Asmaz E.D., Seyidoglu N. (2022). The prevention role of Spirulina platensis (Arthrospira platensis) on intestinal health. Food Sci. Hum. Wellness.

[114] Sayed A.E.-D.H., Hamed M., El-Sayed A.A.A., Nunes B., Soliman H.A.M. (2023). The mitigating effect of Spirulina (Arthrospira platensis) on the hemotoxicity of gibberellic acid on juvenile tilapia (Oreochromis niloticus). Environ. Sci. Pollut. Control Ser..

[115] Ratucoreh C.Y., Retnoaji B. (2018). The growth and histology structure of Indonesian eel (Anguilla bicolor bicolor McClelland, 1844) fed with microalgae. AIP Conference Proceedings. AIP Publishing.

[116] Abu-Zeid E.H., Fattah D.M.A., Arisha A.H., Ismail T.A., Alsadek D.M., Metwally M.M., El-Sayed A.A., Khalil A.T. (2021). Protective prospects of eco-friendly synthesized selenium nanoparticles using Moringa oleifera or Moringa oleifera leaf extract against melamine induced nephrotoxicity in male rats. Ecotoxicology and environmental safety.

